# Clinical interval and diagnostic characteristics in a cohort of bladder cancer patients in Spain: a multicenter observational study

**DOI:** 10.1186/s13104-017-3024-8

**Published:** 2017-12-07

**Authors:** Xavier Bonfill, María José Martinez-Zapata, Robin W. M. Vernooij, María José Sánchez, María Morales Suárez-Varela, Javier De la Cruz, José Ignacio Emparanza, Montserrat Ferrer, José Ignacio Pijoan, Joan Palou, Stefanie Schmidt, Eva Madrid, Víctor Abraira, Javier Zamora, on behalf of the EMPARO-CU study group, Xavier Bonfill Cosp, Mª José Martinez-Zapata, Alborada Martínez, Enrique Morales Olivera, Esther Canovas, Laura Muñoz, Gemma Mas, René Acosta, Ekaterina Popova, Irma Ospina, Mª José Velázquez, Tamara Ruiz Merlo, Gael Combarros Herman, Judit Tirado Muñoz, Robin W.M. Vernooij, Victor Abraira, Javier Zamora, Albert Frances, Carola Orrego Villagran, Rosa Suñol, Dimelza Osorio, Gemma Sancho Pardo, Ignasi Bolívar, José Pablo Maroto, Mª Jesús Quintana, Cristina Martin Lorente, Ferran Algaba, Joan Palou Redorta, Salvador Esquena, Jordi Bachs, Montserrat Ferrer Fores, Stefanie Schmidt, Olatz Garin, Virginia Becerra Bachito, Yolanda Pardo, Amaia Martínez Galarza, José Ignacio Pijoán Zubizarreta, David Manuel Castro Diaz, Juan Manuel Ramos Goñi, Julio López Bastida, Armando Suárez Pacheco, Cesar García López, José Manuel Cozar Olmo, Carmen Martínez, Daysy Chang Chan, Mª José Sánchez Pérez, Ana Isabel Díaz Moratinos, Angel Montero Luis, Asunción Hervás, Carmen Vallejo Ocaña, Costantino Varona, Javier Burgos, Javier Zamora, Jose Alfredo Polo Rubio, Luis López-Fando Lavalle, Miguel Angel Jiménez Cidre, Muriel García Alfonso, Nieves Plana Farras, Rosa Morera Lopez, Sonsoles Sancho Garcia, Victor Abraira, Victoria Gómez Dos Santos, Agustín Gómez de la Cámara, Javier De la Cruz, Juan Passas Martínez, Humberto García Muñoz, Mª Ángeles Cabeza Rodríguez, Irune Ruiz Díaz, José Ignacio Emparanza, Juan Pablo Sanz Jaka, Agustín Llopis González, María Morales, Carlos Camps, Cristina Caballero Díaz, Emilio Marqués Vidal, Francisco Sánchez Ballester, Joaquín Ulises Juan Escudero, Jorge Pastor Peidro, José López Torrecilla, Mª Macarena Ramos Campos, Miguel Martorell Cebollada

**Affiliations:** 1 CIBER de Epidemiología y Salud Pública (CIBERESP), Barcelona, Spain; 2 Iberoamerican Cochrane Centre, Institute of Biomedical Research Sant Pau (IIB Sant Pau), Barcelona, Spain; 3 Public Health and Clinical Epidemiology Service, Hospital de la Santa Creu i Sant Pau, Universitat Autònoma de Barcelona, Barcelona, Spain; 4 Escuela Andaluza de Salud Pública, Instituto de Investigación Biosanitaria de Granada, Barcelona, Spain; 5 Unit of Public Health and Environmental Care, Department of Preventive Medicine, University of Valencia, Valencia, Spain; 6 Hospital 12 de Octubre, Madrid, Spain; 7 Clinical Epidemiology Unit, Hospital Universitario Donostia, BioDonostia, San Sebastian, Spain; 8 Health Services Research Group, IMIM (Hospital del Mar Medical Research Institute), Barcelona, Spain; 9 Clinical Epidemiology Unit, Hospital, Universitario Cruces. Biocruces, Barakaldo, Spain; 10 Fundació Puigvert, Barcelona, Spain; 11 Universitat Autònoma de Barcelona, Barcelona, Spain; 12 Iberoamerican Cochrane Centre, Barcelona, Spain; 13 Biomedical Research Centre-Universidad de Valparaiso-Chile, Valparaiso, Chile; 14 Department of Public Health-School of Medicine, Universidad de Valparaiso-Chile, Valparaiso, Chile; 15 Unidad de Bioestadística Clínica, Hospital Universitario Ramón y Cajal, IRYCIS, Madrid, Spain; 16 Barts and the London School of Medicine and Dentistry, Queen Mary University London, London, UK

**Keywords:** Urinary bladder neoplasm, Neoplasm staging, Time factors, Diagnostic techniques and procedures, Observational study

## Abstract

**Objective:**

We performed a cohort study in seven hospitals in Spain to determine the clinical characteristics of incident patients with bladder cancer, the diagnostic process, and the conditions that might affect health care interval times.

**Results:**

314 patients with bladder cancer were included, 70.3 (Standard Deviation [SD] 11.2) years old and 85.0% male. Clinical stage was T1 in 45.9% of patients. The median interval time between first consultation and diagnosis was of 104.0 days (Inter quartile range [IQR]:112.0; range from 0 to 986), being shorter for those patients who attended a hospital for their first consultation. The median interval time between diagnosis and first treatment was of 0.0 days (IQR: 0.0; range from 0 to 366), being longer when the patient had a pathologic tumor stage ≥ T2a.

**Electronic supplementary material:**

The online version of this article (10.1186/s13104-017-3024-8) contains supplementary material, which is available to authorized users.

## Introduction

Bladder cancer is the ninth most common diagnosed cancer worldwide, contributing with 429,793 new cases yearly [1]. In Spain it is the fifth most common diagnosed cancer with 13,789 new cases yearly [1, 2]. In 2012, the estimated number of deaths due to bladder cancer was of 165,068 worldwide and 5007 in Spain, making it the twelfth leading cause worldwide and the sixth in Spain [1].

Bladder cancer is one of the malignant tumors where a large proportion of health resources are being allocated due to its increasing survival rates and lifelong routine monitoring which involves associated treatment costs, and high recurrence rates [3–6].

Some international initiatives have been undertaken to obtain trustworthy information regarding the healthcare process for bladder cancer patients [7, 8]. In Spain, several studies reported information from hospital minimum data sets and hospital-based cancer registries [9, 10]. These sources of information, however, are quite limited in describing the diagnostic processes, therapeutic approaches, and prognostic factors in bladder cancer. One study conducted in Spain, estimated the annual incidence of bladder cancer and described the clinical profile of patients with bladder cancer, but did not assess the diagnostic and therapeutic processes and potential factors influencing time intervals [2]. For these reasons, the objective of the present study was to examine the clinical care process and health outcomes in incident cases of bladder cancer. In a future article, we will report the results related to the clinical follow-up.

## Main text

### Methods

We performed a multicenter, cohort study of bladder and prostate cancer, in Spain [11]. The research ethics committee from each of the seven tertiary participating hospitals (Additional file 1) approved the protocol. Patient recruitment was done from October 2010 to September 2011. Consecutive patients were selected from the urology and oncology departments and the inclusion criteria were: (1) being diagnosed of bladder cancer during the study period; (2) being diagnosed and treated at one of the participating hospitals; and (3) agree to participate and sign the informed consent form.

**Fig. 1:**
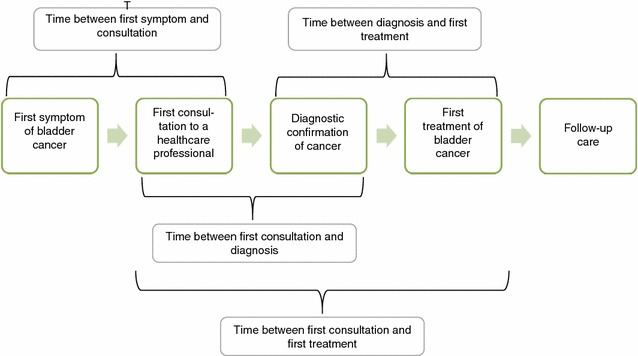
Time intervals considered in our study

Clinical information was gathered by reviewing the medical records and structured interviews to patients (Additional file 2). The outcomes of interest were: socio-demographic data, body mass index (BMI), Charlson index, ECOG WHO score, setting of the first consultation, tests performed to diagnose bladder cancer, pathological results of bladder biopsy, patient tumor clinical stages, and time length of diagnostic and therapeutic intervals (Fig. 1). The time length from first symptoms to first consultation was defined as the period between the date of appearance of the first symptom related to bladder cancer and the date of attendance to the first medical visit (coded as less than 1 month, between 1 and 12 months, after 12 months), which then led to a bladder cancer diagnosis. For asymptomatic patients, the first consultation date was determined by the date when the bladder biopsy was performed. We took the positive biopsy report as the confirmatory diagnosis of the disease, whose date was used to calculate the diagnostic interval. The therapeutic interval was defined as the period between the dates of the pathological diagnosis and the initiation of the first treatment. For categorical variables we calculated relative frequencies; and for continuous variables, the mean and standard deviation (SD) or median and interquartile range (IQR) if skewed variables.

We assessed the association between time variables and potential predictors by using multilevel logistic regression models (patients at first level and hospitals at second level). As potential predictors we considered the following variables: age, BMI, gender, educational level, ECOG WHO score, setting of the first consultation, primary tumor clinical stage, and time since appearance of first symptoms. Continuous time variables were transformed into dichotomous variables. Based on previous studies we established an interval of 100 days as the optimal diagnostic interval, and 30 days for the optimal treatment interval [12–14]. Firstly, an empty model was adjusted considering only the random effect of hospital’s location based on the variability of the two outcomes investigated. Univariate models where then adjusted for each potential predictor. The final model was fitted through a backward selection procedure based on the Wald tests results. Both the empty model and the final multilevel models were estimated by maximum likelihood using the adaptive Gaussian quadrature approximation (with seven quadrature points) [15].

The effect measure was the odds ratio (OR) with 95% confidence interval, and was considered statistically significant if p < 0.05. We calculated the intra-cluster correlation coefficient (ICC) and the median odds ratio (MOR) to estimate the random inter-hospital variability. A lower ICC indicates a lower probability of patients sharing similar hospital experiences. The MOR is the increased risk of moving a patient to a hospital with longer diagnostic and therapeutic intervals [16]. The statistical software used was SPSS, v20.0 (SPSS INC., Chicago, IL, United States of America) and Stata, v12.1 (College Station, TX: StataCorp LP).

### Results

Of the 347 patients recruited, 314 patients participated in the study and 33 were excluded for not meeting the inclusion criteria. Mean age was 70.3 years (SD: 11.2), 267 (85.0%) were male, 194 (61.9%) had at least completed primary studies, and 216 (68.8%) were retired (Table 1). The mean BMI was 27.2 (SD: 4.8) and 180 (57.3%) patients were full active according to the ECOG WHO performance status. The Charlson co-morbidity index was between one and three for 284 patients (90.5%). First consultation for bladder-related symptoms was performed in primary care settings for 151 participants (48.1%), and in hospital settings for the remaining 138 patients (43.9%). In 9.8% of the patients, the disease was diagnosed during a routine visit; in these cases, patients did not report symptoms, or only a certain degree of discomfort caused by the bladder cancer. From the total group, 85.0% were symptomatic; being hematuria the most frequent symptom (73.9%). The time from the first symptoms to first consultation was between 1 month and 1 year for 60.5% of the participants. The most common pathologic malignant diagnosis was urothelial cell carcinoma (90.1%). Other pathologic diagnoses were adenocarcinoma (8.6%), and squamous cell carcinoma (0.6%). The most frequent primary tumor clinical stage was T1 (45.9%).

**Table 1:** Characteristics of bladder cancer patients

Variables	N = 314, n (%)/x ± SD
Mean age ± SD (years)	70.3 ± 11.2
Missing (%)	1 (0.3)
Mean BMI ± SD (Kg/m^2^)	27.2 ± 4.8
Missing	8 (2.5)
Sex	
Male	267 (85.0)
Female	47 (15.0)
Missing	0 (0.0)
Working status	
Active	48 (15.3)
Sick leave	16 (5.1)
Retired	216 (68.8)
Unemployed	11 (3.5)
Other	21 (6.7)
Missing	2 (0.6)
Education	
No education	40 (12.8)
Incomplete primary education	73 (23.2)
Primary education	52 (16.6)
Graduate school	66 (21.0)
Upper secondary studies	36 (11.5)
University	40 (12.8)
Missing	7 (2.2)
ECOG WHO score	
Fully active	180 (57.3)
Restricted	106 (33.8)
Unable to work/only self-care activities/bedridden	26 (8.3)
Missing	2 (0.6)
Setting first consultation	
Primary care	151 (48.1)
Hospital	138 (43.9)
Other	15 (4.8)
Missing	10 (3.2)
Symptoms	
No symptoms or discomfort	47 (15.0)
One or more symptoms	267 (85.0)
Missing	0 (0.0)
Charlson index	
1–3	284 (90.5)
4	9 (2.9)
≥ 5	21 (6.6)
Start of first symptoms including patients with discomfort before first consult	
Up to 1 month	52 (16.6)
Between 1 month and 1 year	190 (60.5)
More than 1 year	53 (16.9)
Missing	19 (6.0)
Primary tumour clinical stage (T)	
Tx	9 (2.9)
Ta	91 (29.0)
Tis	8 (2.6)
T1	144 (45.9)
T2a–b	50 (15.9)
T3a–b	7 (2.3)
T4a–b	4 (1.3)
Missing	1 (0.1)
Node stage (N)	
Nx	88 (28.0)
No	213 (67.9)
N1	6 (2.0)
N2	6 (2.0)
N3	1 (0.1)
Missing	0 (0.0)
Metastasis stage (M) (%)	
Mx	0 (0.0)
M0	303 (96.5)
M1	11 (3.5)
Missing	0 (0.0)
Median interval time between first consultation and diagnosis in days ± IQR (range)	104.0 ± 112.0 (from 0 to 986)
Missing	7 (2.3)
Median interval time between diagnosis and first treatment ± IQR (range)	0.0 ± 0.0 (from 0 to 366)
Missing	0 (0.0)
Median interval time between first consultation and first treatment ± IQR (range)	109.0 ± 120.7 (from 0 to 986)
Missing	6 (1.9)

A bladder ultrasound was reported in 79.0% of the patients and a cystoscopy in 52.2% (Additional file 3). The median diagnostic time interval was 104.0 days (IQR: 112.0) (Table 1). A statistically significant variability was found among hospitals for this interval (MOR: 1.47, 95% CI: 1.14–3.06) (Table 2). Patients who went to primary care setting presented an OR of 1.64 (95% CI 1.03–2.63, p = 0.038) of having a diagnostic interval longer than 100 days compared to patients who were first attended at the hospital. Furthermore, patients who experienced first symptoms longer than 1 month presented an OR of 2.38 (95% CI 1.25–4.51, p = 0.008) of having a diagnostic interval longer than 100 days compared to patients who experienced their symptoms in less than 1 month. There were no significant differences in terms of gender, age, BMI, educational level, ECOG WHO score, or primary tumor stages (Table 2; Additional file 4). The multivariate analysis did not show statistically significant variability among hospitals for this time interval.

**Table 2:** Time intervals and potential determinants

Hospital random effect empty model	Time interval between first consultation and first diagnosis					Time interval between diagnosis and first treatment				
			Empty model ICC/MOR 0.05/1.47	95% CI MOR 1.14–3.06	P-value 0.037			Empty model ICC/MOR 0.26/2.81	95% CI MOR 1.56–11.06	P-value < 0.001
	Median (days)	IQR (days)	OR > 100 days	95% CI OR	P-value	Median (days)	IQR (days)	OR > 30 days	95% CI OR	P-value
Univariate regression										
Gender										
Male	104	113	1			0	366	1		
Female	108.5	104	1.11	(0.58–2.10)	0.760	0	60	1.31	(0.44–3.87)	0.630
Age										
< 65	104	105	1			0	203	1		
≥ 65	107.5	114	0.99	(0.61–1.62)	0.976	0	366	0.99	(0.40–2.46)	0.984
BMI										
< 25	90	99	1			0	364	1		
≥ 25	110	116	1.39	(0.83–2.32)	0.211	0	366	0.39	(0.16–0.94)	0.037
Education level										
Primary education or lower	102.5	107	1			0	366	1		
Graduate school or higher	110	119	1.39	(0.86–2.24)	0.174	0	364	1.18	(0.49–2.85)	0.714
ECOG WHO score										
Fully active	103.5	115	1			0	364	1		
Restricted or worse	111	110	1.29	(0.80–2.06)	0.295	0	366	0.78	(0.32–1.91)	0.586
Specialist first consultation*										
Primary care	115	116	1			0	364	1		
Hospital or specialist	91.5	104	0.61	(0.38–0.97)	0.038	0	366	0.99	(0.41–2.41)	0.981
Primary tumour clinical stage^										
T1	110	109.5	1			0	366	1		
T2a–T4	102	120	0.78	(0.44–1.40)	0.404	0	364	4.39	(1.72–11.21)	0.002
Time since first symptom										
Up to 1 month	56.5	103	1			0	366	1		
More than 1 month	110.5	110	2.38	(1.25–4.51)	0.008	0	364	1.03	(0.32–3.34)	0.959

	Final model ICC/MOR	95% CI MOR	P-value	Final model ICC/MOR	95% CI MOR	P-value				
Multivariate regression										
Hospital random effect	0.05/1.48	1.12–3.78	0.078	0.29/3.06	1.63–13.1	< 0.001				

*BMI* body mass index,* IQR* interquartile range,* SD* standard deviation,* OR* odds ratio,* ICC* intra-cluster correlation coefficient,* MOR* median odds ratio

*  In the multivariate analysis to consult first Hospital or specialist shortened the time to diagnosis compared to consult Primary Care, OR 0.61 [0.38–0.97]

^^^In the multivariate analysis the clinical stage T2a–T4 lengthened the time to treatment compared with clinical stage T1, OR 4.39 [1.72–11.21]

The median therapeutic interval was 0.0 days (IQR: 0.0) (Table 1). There was a statistically significant variability among hospitals for this interval (MOR: 2.81, 95% CI 1.56–11.06, p < 0.001). Patients with a BMI ≥ 25 showed a significant lower odd of having a therapeutic interval longer than 30 days (OR = 0.39; 95% CI 0.16–0.94, p = 0.037). Patients in a tumor stage from T2a–T4b presented an OR of 4.39 (95% CI 1.72–11.21, p = 0.002) of having a therapeutic interval longer than 30 days compared to patients with inferior clinical stages. No significant differences were found within the other outcomes. The multivariate analysis showed statistically significant variability among hospitals in the therapeutic interval and the only factor that significantly influenced this interval was the tumor stage (Table 2).

### Discussion

Our multicenter study in Spain included 314 patients mostly diagnosed with bladder urothelial cell carcinoma. The population characteristics were similar to those described in previously published studies [2, 17–21]. The majority of bladder cancers started with symptoms, being hematuria the most frequent. The percentage (42.3%) of localized tumors was similar to another study conducted in Spain [2], but considerably higher than other previous studies [17, 21].

Most patients in our study population had an early stage of bladder cancer and the diagnosis interval was relatively long, with a median of 104.0 days. Patients who experienced first symptoms for no longer than 1 month before the first consultation and those who went to a hospital for their first consultation had a significantly narrower diagnostic interval; this was expected as the hospital has the possibility of performing TUR (TransUrethral Resection) and biopsy (diagnosis of certainty and treatment), and in primary care only basic imaging tests. There was not significant variability among hospitals in relation to this time interval. The delay in diagnostic interval is concordant with other studies [22, 23], and consequently some European initiatives have emerged to narrow this interval [24–26]. These initiatives suggest that expediting the initial ultrasonography/cystoscopy in all patients could improve the time of diagnosis and treatment for bladder cancer. The European Association of Urology guideline recommends ultrasound as one of the initial staging techniques for patients with hematuria, and cystoscopy only for patients experiencing symptoms suggestive of bladder cancer [27].

Previous Spanish studies assessed the diagnostic and therapeutic time intervals in cancer patients, however they were mainly performed in a single hospital and they all included other types of cancer [28, 30], except for one conducted more than 20 years ago [29]. One Spanish multicenter study reported a mean treatment interval longer than ours (73.2 days) due to a different definition of this variable [13]. They measured the time from the first performed diagnostic tests and not from the date of histological confirmation of bladder cancer until treatment.

In addition, our results reveal that among centers there is a significant variability in terms of treatment intervals. Diverse population characteristics, health care organizations (e.g. health care pathways connecting primary and specialized care) and clinical policies across the different Spanish regions may explain, at least in part, this observed heterogeneity.

Some patients’ and tumor features were also associated with the length of time between diagnosis and treatment; e.g. among patients with a higher primary tumor clinical stage. This finding may be explained because this group of patients is generally treated with cystectomy and chemotherapy and require a more complex process than a TUR approach.

## Limitations

This study may be prone to some limitations. Information bias is a potential issue as the study is based exclusively on information obtained from hospital clinical records. As a result, some outpatient factors, such as those related to consultations in primary care settings, may not have been properly forethought.

## Additional files

**Additional file 1.** List of Ethic Committees that approved the study.

**Additional file 2.** Patients’ structured interview.

**Additional file 3.** Diagnostic tests for bladder cancer patients.

**Additional file 4.** Characteristics of bladder cancer patients by hospitals.

## Abbreviations

BMI: body mass index
ECOG: Eastern Cooperative Oncology Group
ICC: intra-cluster correlation coefficient
IQR: interquartile range
MOR: median odds ratio
OR: odds ratio
SD: standard deviation
USA: United States of America
USD: United States dollar
WHO: World Health Organization
