# Supplementary material for: Clinical interval and diagnostic characteristics in a cohort of bladder cancer patients in Spain: a multicenter observational study
Source: BMC Res Notes. 2017 Dec 7;10:708. doi: 10.1186/s13104-017-3024-8 (PMC5719559; doi:10.1186/s13104-017-3024-8)
Supplement: Supplementary file 1 — Additional file 1. List of Ethic Committees that approved the study. [file 13104_2017_3024_MOESM1_ESM.docx]

Additional file 1: List of Ethic Committees that approved the study

| Hospital de la Santa Creu i Sant Pau (Barcelona) |
| --- |
| Fundación Puigvert (Barcelona) |
| Hospital 12 de Octubre (Madrid) |
| Hospital Ramón y Cajal (Madrid) |
| Basque Country Ethics Committee  Hospital Universitario Donostia (San Sebastián)  Consorcio Hospital General Universitario de Valencia  Hospital Nuestra Señora del Mar (Barcelona)  Hospital Virgen de las Nieves (Granada) |
