# Supplementary material for: Clinical interval and diagnostic characteristics in a cohort of bladder cancer patients in Spain: a multicenter observational study
Source: BMC Res Notes. 2017 Dec 7;10:708. doi: 10.1186/s13104-017-3024-8 (PMC5719559; doi:10.1186/s13104-017-3024-8)
Supplement: Supplementary file 2 — Additional file 2. Patients’ structured interview. [file 13104_2017_3024_MOESM2_ESM.docx]

Additional file 2: Patients’ structured interview

**Could you explain us how your illness began? (Mark if yes)**

**Specify the first signs or symptoms of the disease**

- - I had no symptoms or discomfort
  - Presence of blood in the urine
  - Increased frequency of urination
  - Urgent urination
  - Pain or burning during urination
  - Persistent pain in the back, hips or pelvis
  - Weight loss
  - Fatigue
  - Inability to retain urine
  - Urinary infection
  - Other discomfort, SPECIFY:

**The onset of the first symptom or sign was:**

- - One month ago
  - Between one month and one year
  - More than a year

**Why did you go to the doctor?**

- - For routine preventive healthcare
  - For a disease control
  - For other reasons, SPECIFY:

**Where did attend on your first consultation?**

- - General Physician at primary health care
  - Primary Care Specialist in the Urology service
  - Emergency primary care
  - Hospital Specialist in the Urology service
  - Hospital’s emergency service
  - Business Private Physician
  - Private Medical Center
  - Others (specify):

Date of consultation:

**Labor status at the onset of illness**

- Active
- Housewife
- Temporary work loss
- Renter
- Definitive work termination
- Unemployed
- Retired
- Others (specify):

**What is your current occupation or the last one you have been?**

(If you have more than one profession, specify where do you work)

**What is or was your employment situation in that occupation?**

- Self-employed without employees
- Self-employed worker with 10 or more employees
- Self-employed worker with less than 10 employees
- Manager of a company with 10 or more employees
- Manager of a company with less than 10 employees
- Foreman, supervisor or manager
- Other employee

Code CNO94:

**Which of the following studies have you completed?**

- He/She does not know how to read or write
- No studies
- Incomplete primary studies
- Primary education
- Primary school graduate
- Secondary school or similar studies
- University degree (expert, technical engineering, university schools or similar)
- Higher university studies (higher engineering, bachelor's or PhD)
