# Supplementary material for: Clinical interval and diagnostic characteristics in a cohort of bladder cancer patients in Spain: a multicenter observational study
Source: BMC Res Notes. 2017 Dec 7;10:708. doi: 10.1186/s13104-017-3024-8 (PMC5719559; doi:10.1186/s13104-017-3024-8)
Supplement: Supplementary file 3 — Additional file 3. Diagnostic tests for bladder cancer patients. [file 13104_2017_3024_MOESM3_ESM.docx]

Additional file 3: Diagnostic tests for bladder cancer patients

| **Diagnostic test** | **N=314**  **n (%)** |
| --- | --- |
| **Ultrasound**  Bladder  Renal  Prostate | 248 (79.0)  159 (50.6)  88 (28.0) |
| **Cystoscopy** | 164 (52.2) |
| **Urine cytology** | 102 (32.5) |
| **Physical Examination** Rectal  Vaginal | 72 (22.9)  5 (1.6) |
| **CT Scan**  Abdominal  Abdominothoracic  Thoracic  Cranial | 36 (11.5)  29 (9.2)  16 (5.1)  3 (1.0) |
| **Scintigraphy** | 11 (3.5) |
| **Pyelography** | 6 (1.9) |
| **Nuclear magnetic resonance**  Abdominothoracic  Thoracic  Abdominal  Cranial | 3 (1.0)  2 (0.6)  2 (0.6)  1 (0.3) |
