# Supplementary material for: Clinical interval and diagnostic characteristics in a cohort of bladder cancer patients in Spain: a multicenter observational study
Source: BMC Res Notes. 2017 Dec 7;10:708. doi: 10.1186/s13104-017-3024-8 (PMC5719559; doi:10.1186/s13104-017-3024-8)
Supplement: Supplementary file 4 — Additional file 4. Characteristics of bladder cancer patients by hospitals. [file 13104_2017_3024_MOESM4_ESM.docx]

Additional file 4: Characteristics of bladder cancer patients by hospitals

| **Centres** | **Hospital A (n=52)** | **Hospital B (n=45)** | **Hospital C (n=10)** | **Hospital D (n=67)** | **Hospital E (n=43)** | **Hospital F (n=12)** | **Hospital G (n=85)** |
| --- | --- | --- | --- | --- | --- | --- | --- |
| **Mean age** ± SD,  Missing (%): | 72.2 ± 10.5  0.0 | 69.7 ± 12.2 2.2 | 66.2 ± 7.6  0.0 | 69.1 ± 11.2  0.0 | 69.3 ± 10.0  0.0 | 71.1 ± 7.7  0.0 | 71.2 ± 12.3  0.0 |
| **Mean BMI** ± SD  Missing (%): | 28.2 ± 5.7  1.9 | 26.1 ± 3.3 0.0 | 24.8 ± 3.6  0.0 | 27.5 ± 4.8  0.0 | 28.7± 4.8  0.0 | 29.6 ± 6.4  0.0 | 26.2 ± 4.3  8.2 |
| **Gender** (% male): Missing (%): | 82.7  0.0 | 82.2  0.0 | 70.0  0.0 | 89.6  0.0 | 83.7  0.0 | 83.3  0.0 | 87.1  0.0 |
| **ECOG WHO Performance status** (%) Fully active:  Restricted or worse:  Missing: | 65.4  34.6  0.0 | 62.2  37.8  0.0 | 100.0  0.0  0.0 | 44.8  55.2  0.0 | 60.5  39.5  0.0 | 25.0  66.7  8.3 | 57.6  41.2  1.2 |
| **Primary tumour clinical stage (T)** (%)  Tx:  Ta:  Tis:  T1:  T2a-b:  T3a-b:  T4a-b:  Missing: | 1.9  34.6  1.9  46.2  13.5  1.9  0.0  0.0 | 6.7  42.2  2.2  28.9  17.7  0.0  2.2  0.0 | 0.0  0.0  0.0  80.0  10.0  10.0  0.0  0.0 | 0.0  0.0  3.0  73.1  20.9  3.0  0.0  0.0 | 0.0  23.3  0.0  65.1  11.6  0.0  0.0  0.0 | 8.3  41.7  0.0  16.7  16.7  8.3  8.3  0.0 | 4.7  45.9  4.7  23.5  15.3  2.4  2.4  1.2 |
| **Node stage (N)** (%)  Nx:  No:  N1:  N2:  N3:  Missing: | 86.5  11.5  0.0  1.9  0.0  0.0 | 4.4  88.9  0.0  4.4  2.2  0.0 | 30.0  60.0  0.0  10.0  0.0  0.0 | 0.0  95.5  3.0  1.5  0.0  0.0 | 69.8  30.2  0.0  0.0  0.0  0.0 | 50.0  41.7  0.0  0.0  0.0  0.0 | 2.4  91.8  4.7  1.2  0.0  0.0 |
| **Metastasis stage (M)** (%)  Mx:  M0:  M1:  Missing: | 86.5  13.5  0.0  0.0 | 2.2  95.6  2.2  0.0 | 30.0  70.0  0.0  0.0 | 4.5  92.5  3.0  0.0 | 65.1  32.6  2.3  0.0 | 50.0  50.0  0.0  0.0 | 3.5  92.9  3.5  0.0 |
| **Median interval time between first consultation and diagnosis in days** ± IQR  Missing (%): | 132.0 ± 96.0  5.8 | 111.0± 182.0  4.4 | 77.5 ± 84.3  0.0 | 102.0 ± 122.0  0.0 | 119.0 ± 99.0  0.0 | 69.0 ± 59.0  8.3 | 102.5 ± 110.5  1.2 |
| **Median interval time between diagnosis and first treatment** ± IQR  Missing (%): | 0.0 ± 0.0  0.0 | 0.0 ± 0.0  0.0 | 0.0 ± 34.8  0.0 | 0.0 ± 0.0  0.0 | 0.0 ± 0.0  0.0 | 0.0 ± 0.0  0.0 | 0.0± 0.0  0.0 |
| **Median interval time between first consultation and first treatment** ± IQR  Missing (%): | 124.0 ± 112.0  5.8 | 111.0 ± 182.0  4.4 | 88.5 ± 109.0  0.0 | 110.0 ± 203.0  0.0 | 119.0 ± 99.0  0.0 | 90.0 ± 71.0  8.3 | 102.0 ± 111.0  0.0 |

BMI: Body mass index, ECOG WHO: Eastern Cooperative Oncology Group World Health Organisation, IQR: interquartile range, SD: Standard deviation.
